# Preoperative and intraoperative factors predictive of complications and stricture recurrence following multiple urethroplasty techniques

**DOI:** 10.1002/bco2.83

**Published:** 2021-03-10

**Authors:** H. E. Kay, P. Srikanth, A. V. Srivastava, A. N. Tijerina, V. R. Patel, N. Hauser, A. A. Laviana, J. S. Wolf, E. C. Osterberg

**Affiliations:** ^1^ Dell Medical School University of Texas Austin TX USA; ^2^ Department of Surgery and Perioperative Care Dell Medical School Ascension Seton Hospital Network Austin TX USA; ^3^ Department of Urology Miller School of Medicine University of Miami Miami FL USA

**Keywords:** complication, lichen sclerosus, stricture recurrence, urethral stricture, urethroplasty

## Abstract

**Objectives:**

To investigate factors predictive of postoperative recurrence and complications in patients undergoing urethroplasty for stricture repair at a single center.

**Patients and methods:**

We retrospectively reviewed the records of 108 men who underwent urethroplasty for urethral stricture disease (USD) at a single center from 2016 to 2020. Demographic data, comorbidities, stricture history including etiology and prior treatments, patient‐reported symptoms, and outcomes data were collected for analysis. Data were analyzed in aggregate, then, stratified by type of urethroplasty performed. Descriptive statistics, univariate analysis, multivariate logistic regression, and intergroup comparisons were completed using STATA, with an alpha value of 0.05 and a confidence interval of 95%.

**Results:**

The median age of our patients was 58 years (interquartile range: 42‐69; range: 29‐83), with a median stricture length of 2.0 cm (interquartile range: 1.0‐4.5; range: 0.5‐10). The most common stricture etiology was iatrogenic (n = 33, 31%) and the most common urethroplasty was anterior anastomotic urethroplasty (n = 38, 35%), followed by buccal mucosal graft (BMG) urethroplasty (n = 35, 32%). Twenty‐four patients (22%) had stricture recurrence. Within the aggregate data, recurrence was significantly predicted by obesity (BMI > 30) (Odds Ratio [OR] 3.2, 95% Confidence Interval [CI]: 1.06‐10), and the presence of postoperative complications (OR 6.3, CI: 1.9‐21). The presence of any postoperative complications within 90 days was significantly predicted by stricture length ≥ 5 cm (OR 3.5, CI 1.09‐12) and recurrence (OR 6.0, CI 1.7‐21).

**Conclusion:**

Despite serving as the most definitive treatment for urethral stricture management, stricture recurrence and postoperative complications are not uncommon after urethroplasty. Obesity and stricture length negatively impact outcomes while a penile stricture location is associated with a lower recurrence rate, though this is not statistically significant.

## 1 INTRODUCTION

Urethral stricture disease (USD) is narrowing of the urethra due to inflammation or trauma to the urethra which causes accumulation of scar tissue within the corpus spongiosum and urethral mucosa. There are multiple etiologies for USD including idiopathic, iatrogenic, traumatic, congenital, and infectious.^1^ Associated symptoms include urinary retention, bladder outlet obstruction, genitourinary pain, recurrent urinary tract infections (UTI), and ejaculatory dysfunction, all of which can negatively impact quality of life.^2^ With prevalence estimates ranging from 229 to 627 per 100 000 men and as high as 0.6% in at‐risk populations,^3^ optimizing management and prevention of urethral strictures is an important, yet, understudied, area of research.

Urethroplasty is the most definitive management of urethral strictures, which involves urethral reconstruction through a variety of techniques that depend on the stricture size and location.^1^, ^4^, ^5^ These include direct excision and primary anastomosis of the strictured area, and/or the use of mucosal grafts or flaps to expand the urethral lumen.^6^ Although urethroplasty has a lower recurrence rate than both urethral dilation and direct vision internal urethrotomy (DVIU) procedures, recurrence and complications are not uncommon. It is difficult to quantify recurrence rates after urethroplasty given the variability of stricture disease, the wide variety of surgical techniques, and the lack of uniformity in describing recurrence. Nevertheless, large systematic reviews and smaller single‐center studies have found that stricture recurrence rates over time range from 10% to 58% of patients after urethroplasty, but these have often been narrow in scope, analyzing only a single urethroplasty technique.^7^, ^8^, ^9^, ^10^ In addition, a number of patients experience postoperative complications such as urinary tract infections, urinary leakage, and wound‐related issues.^11^ Thus, despite the favorable outcomes for urethroplasty, there may be independent preoperative and perioperative factors that predict recurrence and complications.

In this retrospective analysis, we sought to investigate factors predictive of postoperative recurrence and complications in patients undergoing multiple urethroplasty techniques for stricture repair at a single center. We hypothesize that independent risk factors for stricture recurrence following urethroplasty include postoperative complications and stricture complexity (i.e., longer length).

## 2 PATIENTS AND METHODS

**FIGURE 1:**
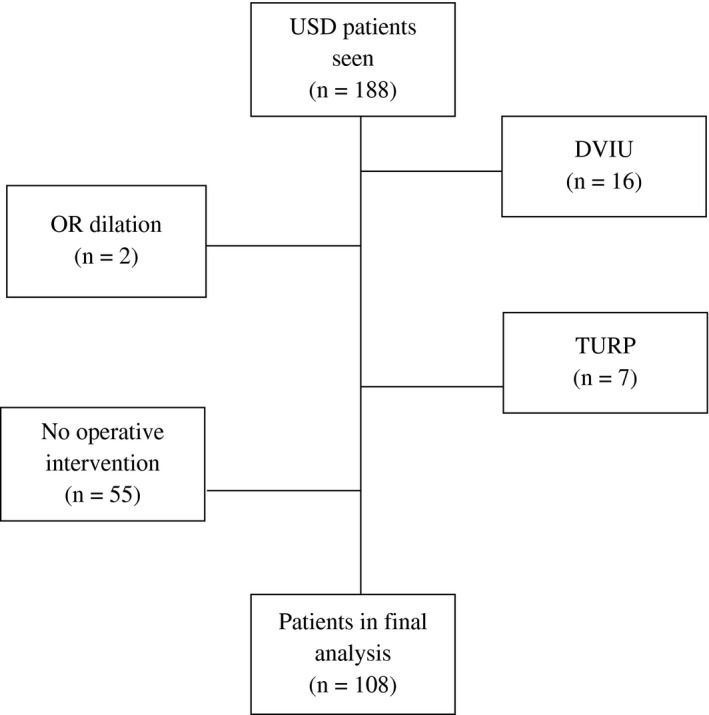
Pathway of patient inclusion and exclusion

We retrospectively reviewed a prospectively gathered database of 188 males who were evaluated for USD by a single surgeon (ECO) at a single tertiary center (Dell Seton Medical Center at the University of Texas, Austin, Texas) from 2016 to 2020. Depending on stricture length and location, patients underwent a variety of surgical procedures for their stricture disease based on surgeon recommendation. Anastomotic urethroplasty, posterior urethroplasty, perineal urethrostomy, and meatoplasty were carried out in relatively standard fashions as previously described.^12^ Substitution urethroplasty (using buccal mucosal grafting [BMG] in dorsal onlay, ventral onlay, and dorsal augmented anastomotic fashions) was carried out as described in the literature.^13^ No skin flaps were used in the penile strictures, and all BMG urethroplasties for penile strictures were dorsal onlay. No ASOPA or inlay techniques were used. Patients were included in our analysis if they underwent one of these operative interventions at our facility for their USD. All patients who did not undergo urethroplasty and those with less than 30 days of postoperative follow‐up at the time of data collection were excluded, reducing our final cohort to 108 men (Figure 1).

Demographic, clinical, and outcomes data were collected for analysis. Age was recorded, and an inflection point of 55 years was used for regression analysis. Comorbidities including diabetes mellitus, obesity, coronary artery disease, prostate cancer, and smoking history were recorded. Stricture history, including etiology (idiopathic, iatrogenic, lichen sclerosus, and traumatic) and prior treatments with DVIU, dilation, and/or prior urethroplasty were recorded. Stricture length and location were reported based on retrograde urethrogram imaging interpretation and/or intraoperative length measurement (cm). Patient symptoms at presentation were assessed objectively with the AUA Symptom score. Recurrent UTI was noted if the patient had two or more UTIs in the last 6 months, or three or more UTIs in the last 12 months. Patient charts were reviewed for postoperative complications up to 90 days after operative intervention and classified according to the Clavien‐Dindo scale.^14^ Urethral stricture recurrence was recorded if the patient described recurrent urinary symptoms and had subsequent cystoscopic or radiologic evidence of recurrence at any point after their operative intervention that required intervention: dilation, DVIU, or repeat urethroplasty. Patients were followed at intervals of 3 and 12 months with Uroflow/post‐void residual measurement(s) and cystoscopy.

A descriptive analysis was performed using variables related to medical history, presenting symptoms, stricture characteristics, and clinical outcomes. Continuous variables were summarized using median and interquartile range and compared using two sample *t* test. Age and stricture length were dichotomized for regression analysis at inflection points of 55 years and 5 cm, respectively. Unadjusted differences were identified using univariate analysis via Chi‐squared test or Fisher's exact test as indicated. Significance was defined with an *P*‐value less than 0.05, and predictors with a *P*‐value < .25 were included in the multivariate logistical regression analysis to control for potential confounders. To assess the performance of the model, calibration was tested with the Hosmer‐Lemeshow (HL) test and discrimination was tested with receiver operating characteristic (ROC) curve analysis. Data were analyzed in aggregate, then, stratified by the type of urethroplasty performed. Statistical analyses were carried out using R‐Studio (Boston, MA, USA) statistical software. Institutional review board permission was granted for this study.

## 3 RESULTS

**FIGURE 2:**
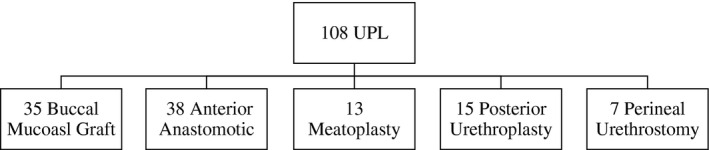
Urethroplasty technique breakdown

Of the 108 men who underwent urethroplasty, anterior anastomotic urethroplasty was the most common approach (n = 38, 35%) followed by BMG urethroplasty (n = 35, 32%) (Figure 2). The age of patients largely followed a Gaussian distribution, with a mean of 55.5 years (median: 58; interquartile range: 42‐69; range: 42‐69). The mean age of men who remained stricture‐free following urethroplasty was 53.8 years versus 61 years in men who had stricture recurrence (*P* < .02). Stricture length followed a nonparametric distribution, with a median of 2.0 cm (interquartile range: 1.0‐4.5; range: 0.5‐10). Strictures etiologies included iatrogenic (n = 33, 31%) idiopathic (n = 27, 25%), trauma (n = 20, 18%), and lichen sclerosus (n = 13, 12%).

(Table S1).

Twenty‐eight patients (26%) experienced one or more postoperative complication(s) within 90 days of surgery. The most frequent complications reported were UTI (n = 7, 25%), wound infection (n = 7, 25%), and obstructed catheter requiring an emergency department visit (n = 5, 17.9%) (Table 1). Twenty‐four patients (22%) had stricture recurrence after urethroplasty at our institution. Time to recurrence ranged from 22 to 442 days, with an average of 138 days. For those with recurrence, 20 had a second operative intervention at our institution––second urethroplasty (n = 2, 1 anterior anastomotic, 1 perineal urethrostomy), meatotomy (n = 2), and urethral dilation (n = 16).

**TABLE 1:** Postoperative complications

	n (% of total)
Scrotal hematoma	2 (1.8%)
Scrotal abscess	1 (0.9%)
Urinary leakage	4 (3.6%)
Sepsis	1 (0.9%)
Obstructed catheter	5 (4.5%)
UTI	7 (6.3%)
Wound infection	7 (6.3%)
Epididymitis	5 (4.5%)

Aggregate stricture recurrence was analyzed with univariate analysis (Table S2), demonstrating significant association with postoperative complications (*P* = .01). On multivariate analysis (Table 2), after controlling for all eligible factors from the univariate analysis, recurrence was significantly predicted by obesity (Odds Ratio [OR] 3.2, 95% Confidence Interval [CI]: 1.06‐10) and postoperative complications (OR 6.3, CI 1.9‐21). Patients with penile (OR 0.21, CI: 0.044‐0.98) strictures were significantly less likely to experience stricture recurrence. Univariate analysis of postoperative complications (Table S3) demonstrated significance with stricture length ≥ 5 cm (*P* = .019) and stricture recurrence (*P* = .012). After multivariate regression analysis (Table 2), stricture length ≥ 5 cm (OR 3.5, CI 1.09‐12) and recurrence (OR 6.0, CI 1.7‐21) remained significant predictors of postoperative complications. All models resulted in HL test values > .69 and c‐Statistic values > .80, indicating good calibration and discrimination, respectively.

**TABLE 2:** Multivariate analysis of factors associated with stricture recurrence or postoperative complication(s)

	Recurrence		Complications	
	Adjusted OR (95% CI)	*P*‐value	Adjusted OR (95% CI)	*P*‐value
*Medical history*				
Obese	3.2 (1.0‐10)	.045*		
*Stricture location*				
Penile	0.21 (0.044‐0.98)	.044*		
Length ≥ 5 cm			3.5 (1.0‐12)	.046*
*Follow‐up*				
Post‐op complication(s)	6.3 (1.9‐21)	.0026*		
Stricture recurrence			6.0 (1.7‐21)	.0042*

* Denotes statistical significance with *P*‐value < .05; *Stricture recurrence*: HL Statistic: 0.923. c‐Statistic: 0.813; *Postoperative complication(s)*: HL Statistic: 0.698. c‐Statistic: 0.805.

Within the BMG group, when controlling for stricture length, location, and prior stricture intervention, patients with penile strictures were less likely to experience stricture recurrence, though the findings were not statistically significant. There were no predictive factors for postoperative complications in this group. Within the anterior anastomotic group, obesity significantly predicted stricture recurrence (OR 1.47, CI 1.12‐1.94). Patients with an indwelling Foley catheter or suprapubic tube at the time of presentation were more likely to experience postoperative complications, however, this was not a significant finding (*P* = .06). There were no significant predictors for recurrence or postoperative complications in posterior urethroplasty, meatoplasty, or perineal urethrostomy cohorts.

## 4 DISCUSSION

Although prior studies have investigated predictors of postoperative complications and recurrence in urethral stricture patients, they are often narrower in scope, investigating only single stricture locations (i.e., bulbar strictures) or single urethroplasty techniques. Due to the size and variance of our patient population, we were able to include variables like stricture length and location among others in our investigation of stricture recurrence. In addition, the inclusion of postoperative complications as a covariate makes this study a more complete analysis of the factors impacting the risk of stricture recurrence. Furthermore, we stratified our data by the most commonly used urethroplasty techniques, allowing for intergroup comparison and analysis. Our findings elucidate the impact of factors like obesity, age, stricture etiology, and urethroplasty type, on univariate and multivariate analysis, as predictors of postoperative complications and stricture recurrence, as detailed below.

First, obesity was a significant predictor of recurrence in both the overall cohort (OR 3.2) and in patients who received an anterior anastomotic urethroplasty (OR 1.47). This is corroborated by evidence on the known perioperative risks and comorbidities associated with a BMI > 30,^15^, ^16^ and this is in line with previous studies. Chapman et al. found obesity to be an independent predictor of stricture recurrence in a study of 596 patients who underwent isolated bulbar urethroplasty.^17^ Breyer et al found that a BMI between 25 and 35 was significantly predictive of urethroplasty failure on univariate analysis of a cohort of 381 patients.^18^ However, neither study was able to investigate obesity on multivariate analysis to isolate its impact away from confounders like diabetes and cardiac disease or compare the impact of obesity across multiple urethroplasty types.

While there is no singular explanation for why obesity may increase perioperative risk, obesity has been associated with systemic chronic low‐grade inflammation, impaired collagen regeneration, and vascular insufficiency, all of which may contribute to urethroplasty failure and stricture recurrence.^19^, ^20^ Obesity may also impair surgical exposure and thus negatively impact the technical feasibility of an operation, particularly in patients with proximal bulbar strictures.^18^ Furthermore, a large suprapubic fat pad can lead to a buried penis and create an environment conducive to chronic inflammation and lichen sclerosus, potentially causing or worsening a urethral stricture.^21^ Interestingly, in Breyer's study, a BMI above 35 was not significantly associated with recurrence, unlike the current study. The authors attribute this to the more sedentary lifestyles of morbidly obese patients, which result in avoidance of activities that impair urethroplasty outcomes.^18^ Given these findings, preoperative counseling on nutrition and weight loss via caloric restriction and low‐impact exercise, may improve intraoperative factors like technical feasibility and operating times, as well as overall urethroplasty outcomes.

Second, patients with postoperative complications were more likely to have stricture recurrence compared to those without postoperative complications, the most common of which were UTI, surgical site infection, and catheter obstruction. It is reasonable to conclude that the resultant immune reaction to any of these complications could compromise healing of the graft site or anastomosis and predispose to urethroplasty failure and stricture recurrence. However, there are few studies that comment on the impact of complications within the 90‐day postoperative period on the risk of stricture recurrence, most likely due to sample size and length of follow‐up. Roehrborn et al. (n = 110, 24% failure rate) found failure rates following urethroplasty to be double when the patient had a positive preoperative urine culture despite antibiotic coverage, but did not include any data on postoperative urine cultures or UTI’s on follow‐up.^22^ With further corroboration, this finding may encourage the use of routine surveillance for signs of postoperative complications like UTI to mitigate the risk of urethroplasty failure.

Third, patients with penile (OR 0.21, CI: 0.044‐0.98) strictures were significantly less likely to experience stricture recurrence, which contrasts with previous studies that cite either no influence of location on recurrence^23^ or that bulbar strictures are less likely to recur.^8^ Because the literature appears to vary so greatly on the impact of stricture location, it is possible that this inconsistency is a result of the inability to control for all known potential confounding variables at this time and across the literature. It is challenging to draw meaningful conclusions from studies of stricture location given the discrepancies across the literature regarding surgical technique, cohort sizes, unaddressed confounding variables, etc.^17^ However, our model is appropriately powered with significant results which is an addition to the literature worth studying further.

Stricture length ≥ 5 cm (OR 3.5, CI 1.09‐12) and recurrence (OR 6.0, CI 1.7‐21) remained significant predictors of postoperative complications. Stricture length is an unsurprising predictor given that longer strictures often require more complex repairs and create a greater surface area for possible recurrence. A 2016 study compared outcomes between dorsal onlay urethroplasty using BMG or penile skin graft (PSG) and found that stricture length had no influence on success rate or functional outcomes, but did not comment on the impact on postoperative complications.^24^ In contrast, other studies have demonstrated that stricture length > 4 cm is associated with stricture recurrence after urethroplasty.^25^ It is interesting to note that our findings relating stricture length to complications were not replicated on subgroup analysis, which likely speaks to the impact of length on choice of technique.

Finally, our model showed that postoperative complications were predicted by stricture etiology related to lichen sclerosus, while no specific variables significantly predicted postoperative complications within the stratified surgical subgroups. The finding related to lichen sclerosus was not statistically significant and likely reflects a low number of patients in our database who presented with lichen sclerosus etiology. Lichen sclerosus (LS) is an inflammatory dermatosis that breaks down the normal architecture of the anogenital skin and urethral mucosa. It often begins in the prepuce and can eventually progress to involve the entire male urethra.^26^ Multiple studies have demonstrated that LS plays a role in stricture recurrence after urethroplasty, regardless of stricture location, length, and repair methodology.^23^, ^27^, ^28^ However, we are unaware of any literature showing a relationship between LS and postoperative complications. We theorize that the presence of lichen changes in the urethra may necessitate more complex surgeries, with increased need for tissue excision to fully remove diseased mucosa, which may explain the increased risk of postoperative complications. These risks should be included in preoperative counseling with patients with stricture due to lichen sclerosus.

On multivariate analysis of surgical technique within the BMG substitution urethroplasty cohort, patients with penile strictures had significantly lower odds of stricture recurrence versus those with other stricture locations (meatus, fossa, bulbar, and membranous) within this cohort. This finding contrasts a previous study of BMG urethroplasty patients citing complex penile stricture as a predictor of recurrence, which is particularly interesting given the similar baseline characteristics between the cohorts with the exception of age (mean age in current study was 58 compared to 44).^29^ Unlike the aforementioned study, we did not characterize the complexity of penile strictures, which may explain the difference. However, alternative studies do endorse BMG urethroplasty as a successful technique for pendulous strictures, without an increase in recurrence rate compared to other anterior stricture locations.^30^ While penile location suggests a protective effect against stricture recurrence in our BMG subgroup model, the finding was not statistically significant.

It is necessary to acknowledge several limitations when interpreting our results. The most notable include a moderate sample size, heterogeneous population, lack of robust patient‐reported outcomes before and after urethroplasty, single‐center series from one surgeon, and incomplete data regarding time to recurrence. Given our sample size, we were not able to sort by specific complication type to observe potential unique relationships, but this is a finding that warrants further investigation. Multiple studies have demonstrated that time after urethroplasty is a key predictive factor in stricture recurrence and longer follow‐up for our series is needed. Breyer et al. had a mean follow‐up time of 5.8 years, with most recurrences occurring in the first 2 years.^18^ Andrich et al. followed a group of 166 patients for 15 years, with a stricture recurrence rate after substitution urethroplasty of 21% at 5 years and 58% at 15 years.^9^ Our subgroup analyses are also underpowered, so it is impossible to draw strong conclusions from the findings as they are presented here.

Despite these limitations, this study is the first comprehensive review of outcomes across multiple urethroplasty types, with the inclusion of predictive variables including demographics, comorbidities, and stricture characteristics. Our results have elucidated several statistically significant predictive factors that could influence urethroplasty technique selection, preoperative patient optimization, patient education, and expectation‐setting prior to urethroplasty. Going forward, we hope to provide the reconstructive surgeon an enhanced understanding of the utility and risk profiles of the multiple techniques in their armamentarium, as well as the predictors for recurrence and complications which may prove valuable in improving overall treatment outcomes and patient satisfaction.

## 5 CONCLUSION

Despite serving as the most definitive treatment for urethral stricture management, stricture recurrence and postoperative complications are not uncommon after urethroplasty. This 4‐year retrospective study establishes predictive factors for complications and recurrence following urethroplasty. Future directions include a focus on patient‐reported outcomes and how stricture recurrence impacts patient satisfaction.

## CONFLICT OF INTEREST

The authors have no other personal or institutional interest with regards to the authorship and/or publication of this manuscript.

## Supporting information

Fig S1

Table S1

Table S2

Table S3
