# Supplementary material for: Preoperative and intraoperative factors predictive of complications and stricture recurrence following multiple urethroplasty techniques
Source: BJUI Compass. 2021 Mar 10;2(4):286–91. doi: 10.1002/bco2.83 (PMC8988843; doi:10.1002/bco2.83)

**Supplemental Figure 1**: Receiver Operator Characteristics (ROC) curves for multivariate logistic regression models of **A)** stricture recurrence (AUC: 0.813, 95% CI: 0.724-0.902. p < 0.001) and **B)** postoperative complications (AUC: 0.805, 95% CI: 0.709-0.901. p < 0.001)


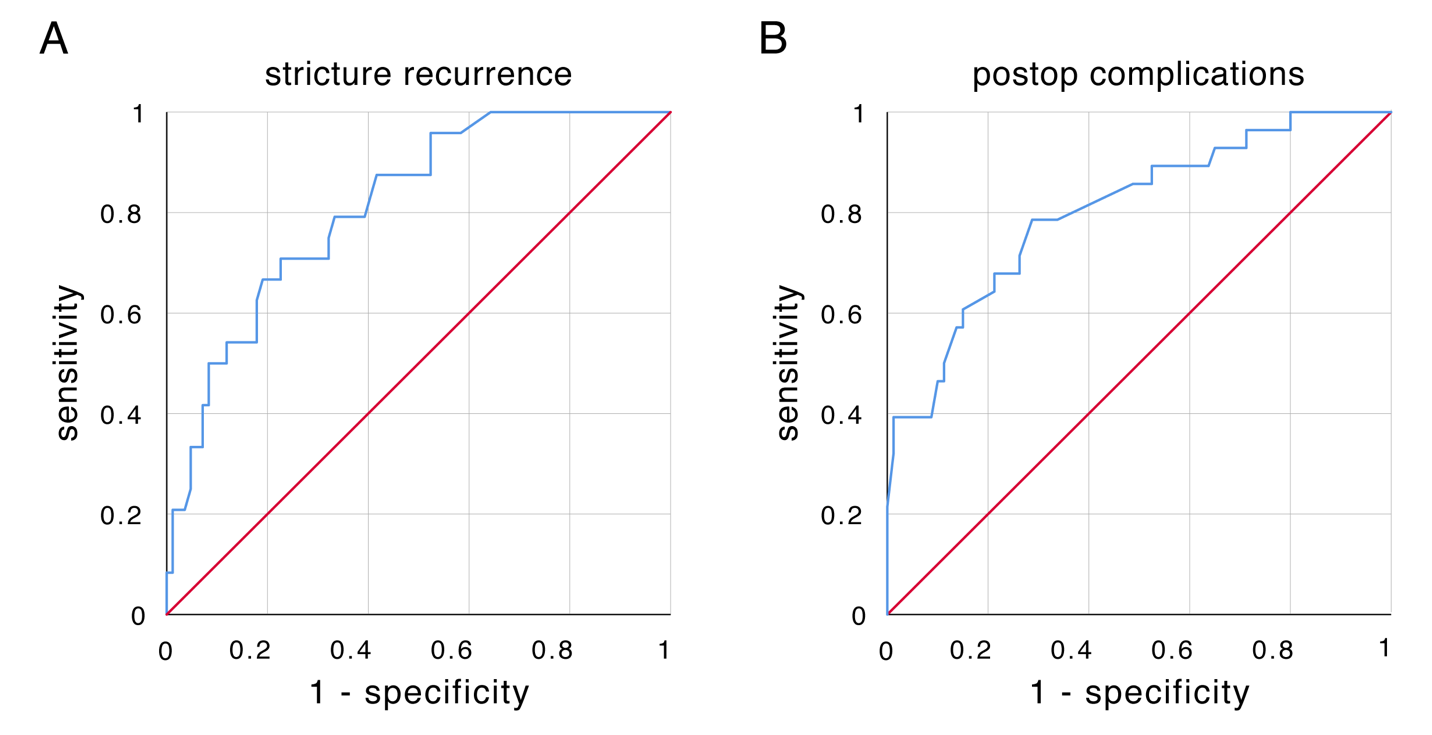

Supplement: Supplementary file 1 — Fig S1 [file BCO2-2-286-s004.docx]
