# Supplementary material for: Preoperative and intraoperative factors predictive of complications and stricture recurrence following multiple urethroplasty techniques
Source: BJUI Compass. 2021 Mar 10;2(4):286–91. doi: 10.1002/bco2.83 (PMC8988843; doi:10.1002/bco2.83)
Supplement: Supplementary file 2 — Table S1 [file BCO2-2-286-s001.docx]

| Supplemental Table 1: Demographic and stricture data in aggregate and by urethroplasty technique | | | | | | | | |
| --- | --- | --- | --- | --- | --- | --- | --- | --- |
|  |  | **Aggregate** | **BMG** | **AA** | **PU** | **Meatoplasty** | **PA** | **p-value** |
| Medical History, n (%) | | | | | | | | |
|  | Obese | 43 (40) | 17 (49) | 15 (39) | 2 (29) | 5 (38) | 4 (27) | 0.74 |
|  | Age ≥55* | 54 (50) | 20 (57) | 10 (26) | 5 (71) | 9 (69) | 10 (67) | <0.001 |
|  | Diabetes | 21 (19) | 8 (23) | 5 (13) | 3 (43) | 3 (23) | 2 (13) | 0.45 |
|  | Abdominal surgery | 35 (32) | 11 (31) | 8 (21) | 3 (43) | 5 (38) | 8 (44) | 0.35 |
|  | Narcotics | 18 (17) | 2 (6) | 7 (18) | 0 (0) | 4 (31) | 5 (33) | 0.038 |
|  | Testosterone | 7 (6) | 3 (9) | 3 (8) | 0 (0) | 0 (0) | 1 (6) | 0.71 |
|  | Ever smoker | 27 (25) | 4 (11) | 7 (18) | 3 (43) | 6 (46) | 7 (44) | 0.075 |
|  | Prostate Cancer* | 11 (10) | 2 (6) | 0 (0) | 0 (0) | 1 (8) | 8 (53) | <0.001 |
|  | Prior TURP | 15 (14) | 6 (17) | 2 (5) | 0 (0) | 5 (38) | 2 (13) | 0.10 |
|  | Prior USD treatment | 78 (72) | 28 (80) | 26 (68) | 6 (86) | 9 (69) | 9 (56) | 0.73 |
| Presenting Symptoms, n (%) | | | | | | | | |
|  | Urgency | 13 (12) | 3 (9) | 5 (13) | 1 (14) | 2 (15) | 2 (13) | 0.72 |
|  | Dysuria | 15 (14) | 5 (14) | 4 (11) | 1 (14) | 3 (23) | 2 (13) | 0.81 |
|  | Hesitancy | 27 (25) | 8 (23) | 10 (26) | 4 (57) | 2 (15) | 3 (20) | 0.31 |
|  | Urinary Retention | 58 (54) | 15 (43) | 20 (53) | 5 (71) | 6 (46) | 12 (80) | 0.11 |
|  | Slow Stream | 72 (67) | 20 (57) | 28 (74) | 5 (71) | 10 (77) | 9 (60) | 0.48 |
|  | Recurrent UTI | 31 (29) | 9 (26) | 11 (29) | 3 (43) | 3 (23) | 5 (33) | 0.91 |
|  | Incontinence* | 11 (10) | 2 (6) | 1 (3) | 1 (14) | 1 (8) | 6 (40) | <0.001 |
|  | Hematuria | 13 (12) | 3 (9) | 5 (13) | 1 (14) | 2 (12) | 3 (20) | 0.95 |
|  | Nocturia | 11 (10) | 2 (6) | 4 (11) | 2 (29) | 1 (8) | 2 (13) | 0.32 |
|  | Current indwelling catheter | 34 (31) | 10 (29) | 12 (32) | 4 (57) | 2 (15) | 6 (40) | 0.12 |
|  | Current self-dilation | 21 (19) | 9 (26) | 2 (5) | 1 (14) | 4 (31) | 5 (33) | 0.038 |
| Stricture Etiology, n (%) | | | | | | | | |
|  | Idiopathic | 27 (25) | 12 (34) | 12 (32) | 0 (0) | 2 (15) | 1 (7) | 0.029 |
|  | Iatrogenic | 33 (31) | 9 (26) | 6 (16) | 3 (43) | 5 (38) | 10 (67) | 0.011 |
|  | Trauma | 20 (18) | 4 (11) | 12 (32) | 1 (14) | 0 (0) | 3 (20) | 0.046 |
|  | BXO* | 13 (12) | 7 (20) | 1 (3) | 1 (14) | 4 (31) | 0 (0) | <0.001 |
| Stricture Location, n (%) | | | | | | | | |
|  | Meatal * | 19 (18) | 3 (9) | 2 (5) | 2 (39) | 12 (92) | 0 (0) | <0.001 |
|  | Fossa | 7 (6) | 2 (6) | 1 (3) | 2 (29) | 2 (15) | 0 (0) | 0.083 |
|  | Penile* | 30 (28) | 21 (60) | 5 (13) | 4 (57) | 0 (0) | 0 (0) | <0.001 |
|  | Bulbar* | 52 (48) | 16 (46) | 33 (87) | 2 (29) | 0 (0) | 1 (6) | <0.001 |
|  | Membranous | 21 (19) | 4 (11) | 3 (8) | 1 (14) | 0 (0) | 13 (87) | <0.001 |
|  | Prostatic* | 16 (15) | 2 (6) | 1 (3) | 0 (0) | 0 (0) | 13 (87) | <0.001 |
|  | Bladder neck | 5 (5) | 2 (6) | 0 (0) | 0 (0) | 0 (0) | 3 (20) | 0.025 |
|  | Panurethral | 1 (1) | 1 (3) | 0 (0) | 0 (0) | 0 (0) | 0 (0) | 0.68 |
|  | Length ≥5cm* | 22 (20) | 21 (60) | 1 (2.6) | 0 (0) | 0 (0) | 0 (0) | <0.001 |
| Follow up, n (%) | | | | | | | | |
|  | Postop complication | 28 (26) | 12 (34) | 9 (24) | 0 (0) | 1 (8) | 6 (40) | 0.30 |
|  | Stricture recurrence | 24 (22) | 7 (20) | 8 (21) | 2 (29) | 2 (15) | 5 (33) | 0.43 |
| *BMG – Buccal mucosal graft; AA – Anterior anastomotic*; *PU – Perineal Urethrostomy; PA – Posterior Anastomotic.*  P-values obtained by ANOVA across the 5 surgical technique groups, excluding aggregate data.  * denotes statistically significant difference with p-value < 0.05 | | | | | | | | |
