# Supplementary material for: Preoperative and intraoperative factors predictive of complications and stricture recurrence following multiple urethroplasty techniques
Source: BJUI Compass. 2021 Mar 10;2(4):286–91. doi: 10.1002/bco2.83 (PMC8988843; doi:10.1002/bco2.83)
Supplement: Supplementary file 3 — Table S2 [file BCO2-2-286-s003.docx]

| **Supplemental Table 2:** Univariate Analysis of Factors Associated with Stricture Recurrence | | | |
| --- | --- | --- | --- |
|  | | **Unadjusted OR (95% CI)** | **p-value** |
| **Medical History** | |  |  |
|  | Age ≥ 55 | 2.4 (0.94 - 6.3) | 0.064 |
|  | Obese | 1.7 (0.68 - 4.3) | 0.25 |
|  | Diabetes | 0.79 (0.24 - 2.6) | 1 |
|  | Abdominal surgery | 0.82 (0.31 - 2.2) | 0.7 |
|  | Narcotics | 1 (0.3 - 3.4) | 1 |
|  | Ever smoker | 1.7 (0.64 - 4.6) | 0.29 |
|  | Prostate Cancer | 1.4 (0.33 - 5.6) | 0.71 |
|  | Prior TURP | 1.3 (0.38 - 4.6) | 0.74 |
|  | Prior USD treatment | 1.2 (0.42 - 3.4) | 0.73 |
| **Presenting Symptoms** | |  |  |
|  | Urgency | 2.5 (0.73 - 8.5) | 0.13 |
|  | Dysuria | 1.3 (0.38 - 4.6) | 0.74 |
|  | Hesitancy | 0.74 (0.25 - 2.2) | 0.59 |
|  | Urinary Retention | 2.5 (0.96 - 6.8) | 0.056 |
|  | Slow Stream | 0.79 (0.31 - 2) | 0.62 |
|  | Recurrent UTI | 1 (0.38 - 2.8) | 0.95 |
|  | Incontinence | 0.76 (0.15 - 3.8) | 1 |
|  | Hematuria | 0.26 (0.032 - 2.1) | 0.29 |
|  | Nocturia | 0.76 (0.15 - 3.8) | 1 |
|  | Current indwelling catheter | 1.2 (0.42 - 3.2) | 0.77 |
|  | Current self-dilation | 2.1 (0.72 - 5.9) | 0.17 |
| **Stricture Etiology** | |  |  |
|  | Idiopathic | 1 (0.35 - 2.9) | 1 |
|  | Iatrogenic | 1.2 (0.45 - 3.1) | 0.74 |
|  | Trauma | 0.56 (0.15 - 2.1) | 0.55 |
|  | BXO | 1.7 (0.46 - 6) | 0.48 |
| **Stricture Location** | |  |  |
|  | Meatal | 1.3 (0.42 - 4.1) | 0.64 |
|  | Penile | 0.3 (0.083 - 1.1) | 0.072 |
|  | Bulbar | 0.45 (0.18 - 1.2) | 0.1 |
|  | Membranous | 2.7 (0.97 - 7.7) | 0.051 |
|  | Prostatic | 1.7 (0.54 - 5.6) | 0.35 |
|  | Bladderneck | 1.8 (0.31 - 11) | 0.61 |
|  | Length ≥ 5 cm | 1 (0.34 - 3.2) | 0.95 |
| **Follow up** | |  |  |
|  | Postop complications | 3.3 (1.3 - 8.7) | 0.012* |
| *denotes statistical significance with p-value <0.05 | | | |
