# Supplementary material for: Preoperative and intraoperative factors predictive of complications and stricture recurrence following multiple urethroplasty techniques
Source: BJUI Compass. 2021 Mar 10;2(4):286–91. doi: 10.1002/bco2.83 (PMC8988843; doi:10.1002/bco2.83)
Supplement: Supplementary file 4 — Table S3 [file BCO2-2-286-s002.docx]

| **Supplemental Table 3:** Univariate Analysis of Factors Associated with Postoperative complication(s) | | | |
| --- | --- | --- | --- |
|  | | **Unadjusted OR (95% CI)** | **p-value** |
| **Medical History** | |  |  |
|  | Age ≥ 55 | 1.5 (0.62 - 3.5) | 0.38 |
|  | Obese | 0.51 (0.2 - 1.3) | 0.16 |
|  | Diabetes | 0.87 (0.29 - 2.6) | 0.81 |
|  | Abdominal surgery | 0.48 (0.17 - 1.3) | 0.15 |
|  | Narcotics | 0.79 (0.24 - 2.6) | 0.78 |
|  | Ever smoker | 0.77 (0.27 - 2.1) | 0.61 |
|  | Prostate Cancer | 1.7 (0.47 - 6.5) | 0.47 |
|  | Prior TURP | 3 (0.97 - 9.2) | 0.048 |
|  | Prior USD treatment | 0.95 (0.36 - 2.5) | 0.91 |
| **Presenting Symptoms** | |  |  |
|  | Urgency | 0.84 (0.21 - 3.3) | 1 |
|  | Dysuria | 0.17 (0.022 - 1.4) | 0.11 |
|  | Hesitancy | 0.57 (0.19 - 1.7) | 0.31 |
|  | Urinary Retention | 0.67 (0.28 - 1.6) | 0.37 |
|  | Slow Stream | 0.47 (0.19 - 1.1) | 0.088 |
|  | Recurrent UTI | 1.6 (0.62 - 3.9) | 0.34 |
|  | Incontinence | 0.61 (0.12 - 3) | 0.72 |
|  | Hematuria | 1.3 (0.37 - 4.7) | 0.74 |
|  | Nocturia | 1.1 (0.27 - 4.4) | 1 |
|  | Current indwelling catheter | 0.88 (0.33 - 2.4) | 0.8 |
|  | Current self-dilation | 1.2 (0.41 - 3.4) | 0.76 |
| **Stricture Etiology** | |  |  |
|  | Idiopathic | 0.57 (0.19 - 1.7) | 0.31 |
|  | Iatrogenic | 1.1 (0.44 - 2.8) | 0.83 |
|  | Trauma | 1.3 (0.44 - 3.8) | 0.65 |
|  | BXO | 2 (0.58 - 6.6) | 0.27 |
| **Stricture Location** | |  |  |
|  | Meatal | 0.29 (0.061 - 1.3) | 0.15 |
|  | Penile | 1.7 (0.66 - 4.2) | 0.28 |
|  | Bulbar | 0.91 (0.38 - 2.2) | 0.83 |
|  | Membranous | 1.6 (0.56 - 4.4) | 0.39 |
|  | Prostatic | 1.4 (0.43 - 4.3) | 0.6 |
|  | Bladderneck | 0.56 (0.062 - 5) | 1 |
|  | Length ≥ 5 cm | 3.1 (1.2 - 8.4) | 0.019* |
| **Follow up** | |  |  |
|  | Stricture recurrence | 3.3 (1.3 - 8.7) | 0.012* |
| *denotes statistical significance with p-value <0.05 | | | |
